# Pediatric obstructive sleep apnea: diagnosis and management

**DOI:** 10.36416/1806-3756/e20240203

**Published:** 2024-07-29

**Authors:** Luiza Fernandes Xavier, Paula Barros de Barros, Sofia Prates da Cunha de Azevedo, Leonardo Araujo Pinto, Magali Santos Lumertz

**Affiliations:** 1. Centro Infant, Escola de Medicina, Pontifícia Universidade Católica do Rio Grande do Sul, Porto Alegre (RS) Brasil.; 2. Programa de Pós-Graduação em Medicina - Pediatria, Escola de Medicina, Pontifícia Universidade Católica do Rio Grande do Sul, Porto Alegre (RS) Brasil.; 3. Serviço de Pediatria, Hospital Moinhos de Vento, Porto Alegre (RS) Brasil.

## INTRODUCTION

Obstructive sleep apnea (OSA) is a respiratory disorder characterized by a reduction in or cessation of airflow in the airways during sleep. It is known to be present in 1-5% of the pediatric population.^1^ ^,^ ^2^ Therefore, OSA is now common in childhood, especially given the significant increase in childhood obesity, which constitutes a significant risk factor for this pathology.^1^ ^,^ ^2^ Another main risk factor is adenotonsillar hypertrophy.^1^ ^,^ ^2^ Consequently, in the pediatric population, OSA is more common in children between two and six years of age.^2^ In addition, prematurity, craniofacial anomalies, neuromuscular diseases, genetic syndromes (such as Down, Prader-Willi, and Crouzon syndromes), asthma, and allergic rhinitis are considered risk factors for the development of pediatric OSA.^1^ ^,^ ^3^ ^,^ ^4^ ^)^

OSA is within the broad classification of obstructive sleep disordered breathing (SDB), which also includes primary snoring, upper airway resistance syndrome, and obstructive hypoventilation.^1^ In addition, OSA is associated with neurocognitive impairment, behavioral problems, failure to thrive, hypertension, and cardiac dysfunction, as well as potentially having systemic repercussions, such as the chronic induction of inflammation, thus contributing to the development of metabolic syndrome and to decreased quality of life.^1^ ^,^ ^2^

## DIAGNOSIS

**Figure 1:**
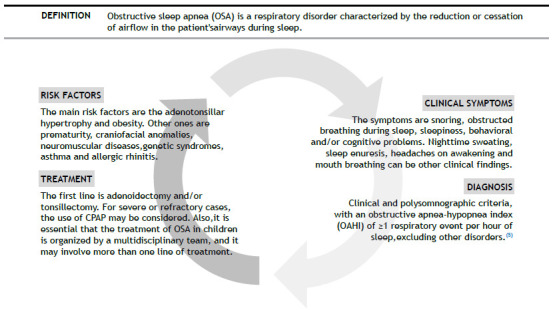
Pediatric Obstructive Sleep Apnea (OSA).

In children, the first step for the clinician is to ask if the child/adolescent snores. An affirmative answer should prompt a more focused evaluation.^2^ Clinical and polysomnography (PSG) criteria that are not attributable to other disorders are necessary for the diagnosis of OSA (Figure 1).^5^

According to the American Academy of Sleep Medicine (AASM), the clinical diagnostic criteria for pediatric OSA include the presence of one or more of the following symptoms^5^: snoring; labored, paradoxical, or obstructed breathing during sleep or drowsiness; hyperactivity; behavioral problems; and learning disabilities or other cognitive problems. Other possible clinical findings of pediatric OSA include night sweats, nocturnal enuresis (especially secondary), headaches on awakening, mouth breathing (during sleep or while awake), tonsillar hypertrophy, adenoidal facies, micrognathia/retrognathia, and a high-arched palate.^2^

The AASM has also established PSG criteria for pediatric OSA,^5^ which include an obstructive apnea-hypopnea index (AHI) ≥ 1 event/hour of sleep, with or without a pattern of obstructive hypoventilation-defined as hypercapnia during ≥ 25% of the total sleep time, together with snoring, flattening of the inspiratory nasal pressure waveform, or paradoxical thoracoabdominal motion. Assessment of the severity of respiratory events according to the PSG findings, even if based on practice and limited consensus, can also help define the management approach. If the respiratory events index, most commonly the AHI, is less than 5 events/hour, OSA is considered mild,^1^ ^,^ ^2^ whereas an AHI of 5.0-9.9 indicates moderate OSA and an AHI ≥ 10 indicates severe OSA.^1^

## MANAGEMENT

The treatment of OSA in children is approached comprehensively, considering their unique needs and their developing physical characteristics. Frequently, it includes more than one option of intervention. Lifestyle modifications, such as promoting weight loss in cases of obesity and establishing consistent sleep routines to ensure a conducive rest environment, are essential considerations for initial therapy. In addition, it might be necessary to treat underlying conditions such as allergies and nasal congestion that contribute to airway obstruction during sleep.^3^

If a patient with pediatric OSA has adenotonsillar hypertrophy and has no contraindication to surgery, surgical removal of the tonsils and adenoids (adenoidectomy and/or tonsillectomy) is the first line of treatment.^1^ ^,^ ^2^ Other specific types of surgery, such as craniofacial surgery (e.g., mandibular distraction osteogenesis), have been proposed in children with syndromic craniofacial abnormalities.^1^ For severe or refractory cases, the use of CPAP may be considered in order to maintain open airways during sleep. The use of nasal corticosteroids, montelukast, or both can be an option in mild cases, as can rapid maxillary expansion and orthodontic appliances in patients with maxillary constriction, retrognathia, or malocclusion.^1^ ^,^ ^2^ It is also essential that the treatment of OSA in children be coordinated by a multidisciplinary team, including pediatricians, otolaryngologists, pulmonologists, and other healthcare professionals, especially when this form of SDB is diagnosed in an infant.^4^ Regular monitoring is necessary in order to assess the efficacy of treatment and adjust it as needed, thus ensuring adequate sleep, promoting healthy growth, and improving the overall well-being of the child.^2^

## PREVENTION AND PROGNOSIS

Clinicians need to be aware of the possibility of OSA symptoms in children and adolescents. The potential consequences of untreated pediatric OSA include neurobehavioral deficits, metabolic alterations, cardiovascular disease (elevated blood pressure, ventricular dysfunction, or pulmonary hypertension), and exacerbation of comorbidities (e.g. asthma), as well as growth impairment.^1^ ^,^ ^2^ ^,^ ^6^ To mitigate the adverse effects of this pathology, preventive measures such as addressing modifiable risk factors can be employed. There are some ways to minimize the risk of SDB: avoiding smoking in the home, treating asthma or allergic rhinitis, assisting with weight reduction (if the child is overweight or obese), and performing surgery for enlarged tonsils and adenoids on the children for whom surgical intervention is indicated.^2^ ^,^ ^3^ ^,^ ^5^
